# Hepcidin and erythroferrone response to 3 weeks of exposure to normobaric hypoxia at rest in trained cyclists

**DOI:** 10.3389/fphys.2023.1279827

**Published:** 2023-11-27

**Authors:** Kamila Płoszczyca, Miłosz Czuba, Małgorzata Chalimoniuk, Konrad Witek, Marcin Baranowski

**Affiliations:** ^1^ Department of Kinesiology, Institute of Sport—National Research Institute, Warsaw, Poland; ^2^ Department of Applied and Clinical Physiology, Collegium Medicum University of Zielona Gora, Zielona Gora, Poland; ^3^ Department of Physical Education and Health in Biala Podlaska, Józef Piłsudski University of Physical Education in Warsaw, Warsaw, Poland; ^4^ Institute of Sport—National Research Institute, Warsaw, Poland; ^5^ Department of Physiology, Medical University of Bialystok, Bialystok, Poland

**Keywords:** hypoxia, live high-train low, hepcidin, erythroferrone, ferroportin, iron, altitude training, athletes

## Abstract

**Purpose:** The effectiveness of altitude training on haematological adaptations is largely dependent on iron metabolism. Hepcidin and erythroferrone (ERFE) are key iron-regulating hormones, yet their response to altitude training is poorly understood. The aim of this study was to analyze changes in hepcidin and ERFE under the influence of 3 weeks of the Live High-Train Low (LH-TL) method.

**Methods:** Twenty male trained cyclists completed a 3-week training program under normoxic conditions (NORM) or with passive exposure to normobaric hypoxia (LH-TL; FiO_2_ = 16.5%, ∼2000 m; 11–12 h/day). Hepcidin, ERFE, hypoxia inducible factor-2 (HIF-2), ferroportin (Fpn), erythropoietin (EPO), serum iron (Fe) and hematological variables were assessed at baseline (S1), then immediately after (S2) and 3 days after (S3) intervention.

**Results:** In the LH-TL group, hepcidin decreased by 13.0% (*p* < 0.001) in S2 and remained at a reduced level in S3. ERFE decreased by 28.7% (*p* < 0.05) in S2 and returned to baseline in S3. HIF-2α decreased gradually, being lower by 25.3% (*p* < 0.05) in S3. Fpn decreased between S1 and S2 by 18.9% (*p* < 0.01) and remained lower during S3 (*p* < 0.01). In the NORM group, in turn, hepcidin levels increased gradually, being higher by 73.9% (*p* < 0.05) in S3 compared to S1. No statistically significant differences in EPO were observed in both groups.

**Conclusion:** Three weeks of LH-TL suppresses resting hepcidin and ERFE levels in endurance athletes. We found no association between hepcidin and ERFE after LH-TL. Probably, ERFE is not the only factor that suppresses hepcidin expression in response to moderate hypoxia, especially in later stages of hepcidin downregulation. With the cessation of hypoxia, favorable conditions for increasing the availability of iron cease.

## Introduction

Altitude/hypoxic training is used in sports to improve exercise capacity. One of the popular methods is the “Live High, Train Low” (LH-TL) procedure, which is likely due to stronger scientific evidence of this approach yielding a sea-level performance benefit as compared to other hypoxic training protocols (Álvarez-Herms et al., 2015). LH-TL involves passive exposure to hypoxia for 10–12 h a day and training in normoxic conditions. To achieve the expected adaptive effects, it is recommended that the procedure should last at least 3 weeks (Czuba et al., 2018; Płoszczyca et al., 2018). One of the key benefits of the LH-TL method is improvement of the blood oxygen carrying capacity by stimulating erythropoiesis. However, the effectiveness of altitude/hypoxic training in this field depends on many factors, including iron stores in the body and regulation of iron transport and metabolism (Płoszczyca et al., 2018). Oxygen transport to tissues is a key factor determining the maximal oxygen uptake (VO_2max_) and aerobic exercise performance of athletes (Wagner, 2007), and it is dependent on several stages: ventilation, diffusion of O_2_ into erythrocytes and its binding by hemoglobin (Hb), blood flow, and O_2_ diffusion from erythrocytes to mitochondria (Mairbäurl, 1994; Wagner, 2007). One step in this pathway is O_2_ transport by Hb. Improvement at this stage can be achieved by an increase in the Hb concentration per unit volume of blood and/or by changes in Hb-O_2_ affinity. Increased Hb concentration can be obtained from adaptation to hypoxic conditions (Płoszczyca et al., 2018).

Exposure to hypoxia leads to the stimulation of erythropoiesis, which is associated with an increase in iron requirements. If iron stores in the body are insufficient or the diet consumed during hypoxic intervention is inadequate for the nutritional demands, hematological variables will not be elevated (Michalczyk et al., 2016; Constantini et al., 2017). It has been shown that in a hypoxic environment, the expression of hepcidin, a key regulator of iron metabolism, is reduced (Hintze and McClung, 2011). Hepcidin works by interacting with the cellular iron exporter, ferroportin (Fpn). Hepcidin binds to Fpn, leading to its internalization and degradation, thus reducing cellular iron export (Nemeth et al., 2004). Lowered hepcidin levels enable increased absorption of dietary iron in the gut and increase iron delivery to the blood plasma. Hepcidin production is regulated in response to changes in circulating iron concentration, iron stores, the iron requirements of erythroid precursors for hemoglobin synthesis or by inflammation (Ganz and Nemeth, 2012). Hepcidin suppression during erythropoiesis is mediated by erythroferrone (ERFE), a hormone which is produced by erythroblasts in response to erythropoietin (EPO) synthesis (Kautz et al., 2014). Both EPO and ERFE increase during hypoxic exposure (Robach et al., 2021). Another key regulator of iron homeostasis is hypoxia inducible factor-2 (HIF-2), which regulates iron absorption via direct transcriptional activation of Fpn (Haase, 2013). HIF-2 is furthermore involved in repressing hepcidin production through the regulation of hepatic EPO expression (Renassiaa and Peyssonnaux, 2019). The activation of this cascade of reactions allows the availability of iron in the body to be increased when doing so is necessary for effective erythropoiesis.

Little research has been done on the response of hepcidin and ERFE to hypoxic exposure and hypoxic training. In several studies, it was observed that during short-term exposure to hypoxia (several hours, several days) there is a decrease in hepcidin and an increase in ERFE levels (Goetze et al., 2013; Emrich et al., 2021; Robach et al., 2021). To our knowledge, only two studies have examined the effect of the LH-TL method on hepcidin and ERFE. Govus et al. (2017) reported that 2 weeks of normobaric hypoxia (200 h at 3000 m) suppressed resting hepcidin levels in athletes. Likewise, hepcidin showed a tendency to decrease in endurance athletes during 3 weeks of LH-TL protocol at 3000 m simulated altitude (Garvican-Lewis et al., 2018). Changes of plasma ERFE, in turn, were not observed during and 7 days after LH-TL in individuals who did not receive iron supplementation (Garvican-Lewis et al., 2018).

LH-TL is a popular training protocol among athletes. The effect of hypoxia on iron metabolism is crucial for the effectiveness of this method, because regulatory mechanisms activated in hypoxia make it possible to cover the increased demand for iron during the period of enhanced erythropoiesis. The aim of our study was to analyze changes in the iron-regulating hormones hepcidin and ERFE in response to 3 weeks of the LH-TL method (FiO_2_ = 16.5%, ∼2000 m; 10–12 h/day) in endurance athletes. We hypothesized that hepcidin would decrease and ERFE would increase under the influence of LH-TL protocol and would remain altered up to 3 days after hypoxic exposure. The level of HIF-2α, EPO, Fpn, and iron (Fe), red blood cell (RBC), hemoglobin (HGB), hematocrit (Hct) and reticulocytes (Ret) were also assessed in an attempt to better understand the iron metabolism regulation and the hematological changes associated with the response to hypoxia.

## Materials and methods

### Participants

Twenty male trained cyclists were recruited for this study. Statistical power analysis (GPower 3.1 software) showed that with such a sample size (n = 20), while maintaining an acceptable power (1-β = 0.80) and *α* = 0.05, the test would detect an effect size (ES) > 0.29 (small). All participants had current medical examinations, without any contraindications that would exclude them from the study. The participants provided written voluntary informed consent before participation. Study participants were randomized to two groups: an experimental group (LH-TL) and control group (NORM). The experimental group (LH-TL) (n = 10; age: 27.1 ± 6.6 years; VO_2max_: 56.3 ± 8.0 mL kg^-1^∙min^-1^; body height 1.84 ± 0.06 m; body mass: 70.8 ± 7.1 kg; body fat content: 7.0% ± 2.2%) was exposed to normobaric hypoxia (FiO_2_ = 16.5%, ∼2000 m) at rest and during sleep for 11–12 h a day. Training in this group was performed under normoxia. The control group (NORM) (n = 10; age: 29.8 ± 4.3 years; VO_2max_: 55.1 ± 5.4 mL kg^-1^∙min^-1^; body height 1.80 ± 0.09 m; body mass: 75.7 ± 8.3 kg; body fat content: 7.5% ± 1.3%) lived and trained under normoxic conditions. The research project was conducted according to the Helsinki Declaration and was approved (No. R-I-002/325/2019) by the Bioethics Committee of the Medical University of Bialystok, Poland.

### Study design

The evaluation included three research series (S1, S2, S3). Each research series was performed after an overnight fast and included venous blood sampling. Additionally, after obtaining the blood samples during S1, participants’ body height, body mass, and body composition were also measured (InBody 220, Biospace, Korea). Next, 2 hours after a light mixed meal (5 kcal/kg of body mass, 50% CHO, 30% Fat, 20% Pro), a graded exercise test (40 W/3 min) was performed using the Excalibur Sport cycle ergometer (Lode, Netherlands) in order to measure VO_2max_ (MetaLyzer 3B-2R, Cortex, Germany), and lactate threshold workload (WR_LT_) based on the Dmax method (Cheng et al., 1992). Our previous studies (Czuba et al., 2009; Płoszczyca et al., 2020) demonstrated that LT determined using the D-max method corresponds to the maximal lactate steady state (MLSS). These data were used to determine an individual training workload for the experiments.

Between S1 and S2, athletes from both groups followed a similar training program for 3 weeks. The training program included 3 weeks with progressive training loads. All the groups followed the same training routines with individually adjusted intensity zones (Table 1). Training load was recorded using power meters (Vector, Garmin). Training load was calculated after each training session and archived using WKO+ 4.0 software (TrainingPeaks, USA). The only factor that differentiated the protocols between groups was the exposure of the LH-TL group to normobaric hypoxia (FiO_2_ = 16.5%, ∼2000 m) by 11–12 h a day (evenings and nights) in a hypoxic training center (Air Zone, Warsaw, Poland) equipped with normobaric hypoxia system AirZone 40 (Air Sport, Poland). The hypoxic dose of 250 h (11–12 h/day) at an altitude of 2000–2500 m (FiO_2_ = 15.5–16.5%), as proposed in the study, has previously been shown to be an effective stimulus leading to improvements in hematological indices (Rusko et al., 2004; Chapman et al., 2014; Czuba et al., 2018). In the LH-TL group, the oxygen saturation of hemoglobin (SpO_2_) was measured on each day after waking up (in lying position) under hypoxic conditions using a Pulsox-3 (Minolta, Netherlands) pulse oximeter (94.9% after week 1; 95.6% after week 2; and 96.1 after week 3).

**TABLE 1:** Training program during the experiment.

Day	Week 1	Week 2	Week 3
1	100% WR_LT_ for 30 min + 2 h endurance training (60%–75% of WR_LT_)	100% WR_LT_ for 35 min + 2 h endurance training (60%–75% of WR_LT_)	100% WR_LT_ for 40 min + 2 h endurance training (60%–75% of WR_LT_)
2	3–4 h of endurance training 60%–75% of WR_LT_ with high-speed intervals (2 × 6 × 10 s-max)	3–4 h of endurance training 60%–75% of WR_LT_ with high-speed intervals (2 × 6 × 10 s-max)	3–4 h of endurance training 60%–75% of WR_LT_ with high-speed intervals (2 × 6 × 10 s-max)
3	100% WR_LT_ for 30 min + 2 h endurance training (60%–75% of WR_LT_)	100% WR_LT_ for 35 min + 2 h endurance training (60%–75% of WR_LT_)	100% WR_LT_ for 40 min + 2 h endurance training (60%–75% of WR_LT_)
4	Strength endurance (gym)	Strength endurance (gym)	Strength endurance (gym)
	Upper body	Upper body	Upper body
5	100% WR_LT_ for 30 min + 2 h endurance training (60%–75% of WR_LT_)	100% WR_LT_ for 35 min + 2 h endurance training (60%–75% of WR_LT_)	100% WR_LT_ for 40 min + 2 h endurance training (60%–75% of WR_LT_)
6	3–4 h of endurance training 60%–75% of WRLT with high-speed intervals (2 × 6 × 10 s-max)	3–4 h of endurance training 60%–75% of WRLT with high-speed intervals (2 × 6 × 10 s-max)	3–4 h of endurance training 60%–75% of WRLT with high-speed intervals (2 × 6 × 10 s-max)
7	Day off	Day off	Day off

WR_LT_, Lactate threshold workload determined under normoxia.

All athletes lived in the same accommodations and followed the same training schedule, sleeping time, and diet. During the experiments, the participants consumed a controlled mixed diet (50% CHO, 20% Fat, 30% Pro). Daily energy intake was set at ∼3500 kcal. The average dietary iron content was 22 ± 1.9 mg per day. Athletes did not receive iron supplementation. The third series of tests (S3) was carried out during the recovery week, 3 days after the completion of the LH-TL procedure.

### Blood sampling and determination of Hepcidin, Erythroferrone, Ferroportin, HIF-2α and Erythropoietin

Blood samples were taken from all participants at three time-points: before the training program, immediately after its completion, and following 3 days of rest. In each case, 10 mL of venous blood was collected from the antecubital vein under fasting conditions between 7:00–8:30 a.m. The blood samples were used to obtain serum after being left at room temperature for 30 min. The serum was frozen and stored at −70°C until analyses. Serum levels of hepcidin, erythroferrone, ferroportin, and HIF-2α were determined by EIA immunoassay using a commercial ELISA Hepcidin (No MBS164980), Erythroferrone (No MBS2707354), Ferroportin (No MBS1603201), and HIF-2α (No MBS771952) kit (My BioSource, San Diego, CA, USA) following the manufacturer’s instructions. Hepcidin, erythroferrone, ferroportin, and HIF-2α assays were performed in two batches, and in the range of 0.312–20 μmol/mL. The minimum detectable dose of hepcidin, erythroferrone, ferroportin and HIF-2α is typically less than 5.12 pg/mL, 0.064 ng/mL, 0.093 ng/mL and 10.0 pg/mL, respectively. The intra-/interassay coefficients of variation were <8–10% and <10–15%, respectively. Serum levels of EPO was determined by Abcam’s Erythropoietin (EPO) Human *in vitro* ELISA kit (No ab119522; Abcam, Cambridge, United Kingdom) following the manufacturer’s instructions. The limit of detection of Human EPO was determined to be 0.17 mlU/mL. The intra-/interassay reproducibility was <4% and <7%, respectively. The hematological markers (RBC, HGB and HCT) were determined by using a Sysmex XN 2000 analyzer (Sysmex Corporation, Kobe, Japan). Reticulocyte counts were measured manually. Serum iron was determined by spectrophotometry using a Cobas 6000/c501 analyzer (Roche Diagnostics, Mannheim, Germany). To exclude the potential influence of hemoconcentration on changes in hematological variables, we measured the total protein concentration in blood, using the BCA protein assay kit (Sigma). Bovine serum albumin (fatty acid free, Sigma) was used as a standard. No significant differences were found between the groups or test series (LH-TL group—66.34 ± 5.38 mg/mL before training, 66.59 ± 7.24 mg/mL after training, 67.15 ± 5.82 3 days after training; NORM group—71.61 ± 7.63 mg/mL before training, 72.10 ± 3.29.24 mg/mL after training, 68.13 ± 5.5.98 3 days after training).

### Statistical analysis

The results of the study were analyzed using StatSoft Statistica 13.0 software (TIBCO Software Inc., Palo Alto, CA, USA). The results were presented as arithmetic means (x) ± standard deviations (SD). The statistical significance level for all the analyses was set at *p* < 0.05. Prior to all the statistical analyses, normality of the distribution of variables was checked using the Shapiro–Wilk test. The analysis of variance (ANOVA) for repeated measures (intervention [LH-TL, NORM] x time [S1, S2, S3]) was used to determine the differences in each of the dependent variables. When significant differences were found, the *post hoc* Tukey test was used. The relationships between hepcidin and ERFE and the other variables were analyzed using Pearson’s correlation coefficient. The effect sizes (ESs) were calculated from standardized differences (Cohen’s d units). The threshold values for Cohen ES statistics were considered to be small (0.20–0.60), moderate (0.60–1.20), large (1.20–2.0), very large (2.0–4.0), or extremely large (>4.0) (Hopkins et al., 2009).

## Results

Statistical analysis showed significant group × time interactions for hepcidin (F = 9.338; *p* < 0.001), ferroportin (F = 6.356; *p* < 0.01) and HIF-2α (F = 7.073; *p* < 0.01) levels. Additionally, the time effect was found for the levels of erythroferrone (F = 5.492; *p* < 0.01), RBC (F = 3.949; *p* < 0.05), HGB (F = 3.761; *p* < 0.05) and Hct (F = 4.870; *p* < 0.05).

**FIGURE 1:**
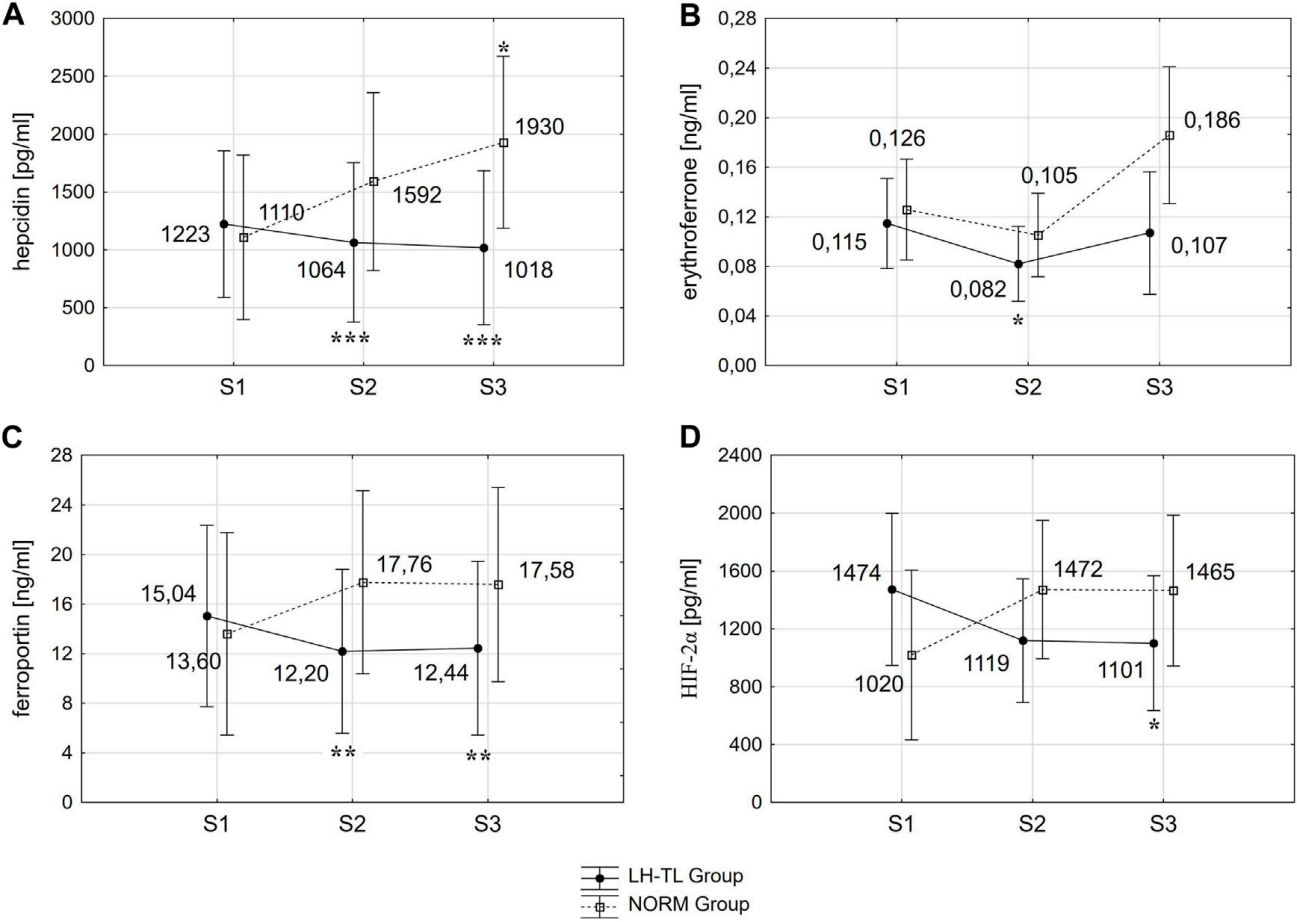
Changes in hepcidin **(A)**, erythroferrone **(B)**, ferroportin **(C)** and HIF-2α **(D)** immediately (S2) and 3 days (S3) after LH-TL (2000 m simulated altitude) and normoxic training. *p < 0.05; **p < 0.01; ***p < 0.001 - statistically significant differences from baseline (S1).

The results revealed that in the LH-TL group hepcidin levels decreased significantly by 13.0% (*p* < 0.001; ES: 0.16) immediately after training (S2) and remained at a reduced level 3 days after cessation of training (S3) (*p* < 0.001; ES: 0.21). In NORM group, hepcidin levels increased gradually, being higher by 73.9% (*p* < 0.05; ES: 0.62) 3 days after training (S3) compared to pre training (S1) (Figure 1A).

ERFE decreased significantly by 28.7% (*p* < 0.05; ES: 0.86) immediately after training (S2) and returned to baseline levels 3 days after training (S3) in the LH-TL group. In the NORM group, ERFE tended to increase 3 days after training (S3) in relation to S2, but this change did not reach statistical significance (*p* < 0.07) (Figure 1B).

Fpn decreased significantly between S1 and S2 by 18.9% (*p* < 0.01; ES: 0.26) and it was still lower during S3 (*p* < 0.01; ES: 0.24) in the LH-TL group. In the NORM group, Fpn levels did not change significantly (Figure 1C).

In the LH-TL group HIF-2α levels decreased gradually, being lower by 25.3% (*p* < 0.05; ES: 0.52) 3 days after training (S3) compared to pre training (S1). In the NORM group the HIF-2α levels did not change significantly (Figure 1D).

RBC, HGB and Hct increased significantly (*p* < 0.05) immediately after LH-TL by 4.6% (ES: 0.72), 5.0% (ES: 0.86) and 4.6% (ES: 0.98), respectively, but 3 days after LH-TL the values did not differ from the baseline. No statistically significant differences in EPO, Fe or Ret were observed after training in any of the groups (Table 2).

**TABLE 2:** Hematological and iron parameters before and after 3 weeks of LH-TL protocol (2000 m simulated altitude) and normoxic training in cyclists.

		S1	S2	S3
		Before training	After training	3 days after training
EPO (mlU/mL)	LH-TL	7.93 ± 1.89	8.57 ± 2.74	7.41 ± 1.69
	NORM	8.34 ± 1.63	8.47 ± 1.63	8.23 ± 1.29
RBC (mln/µL)	LH-TL	4.99 ± 0.26	5.22^a^ ± 0.37	5.06 ± 0.29
	NORM	4.55 ± 0.32	4.59 ± 0.33	4.53 ± 0.25
HGB (g/dL)	LH-TL	14.86 ± 0.77	15.60^a^ ± 0.98	15.20 ± 0.74
	NORM	13.87 ± 0.70	13.98 ± 0.80	13.86 ± 0.74
HCT (%)	LH-TL	43.44 ± 1.34	45.43^a^± 2.19	44.55 ± 1.73
	NORM	40.66 ± 1.89	41.14 ± 2.15	40.74 ± 1.80
Ret (‰)	LH-TL	11.84 ± 2.89	13.55 ± 3.42	13.43 ±4.07
	NORM	14.22 ± 6.38	17.44 ± 7.80	15.00 ± 4.03
Fe (μg/dL)	LH-TL	101.67 ± 44.51	106.30 ± 52.35	117.89 ± 48.60
	NORM	113.67 ± 42.29	95.10 ± 28.03	99.21 ± 48.18

^a^ *p* < 0.05—statistically significant difference from baseline (S1).

Hepcidin significantly correlated with HGB level immediately after LH-TL (r = −0.749; *p* < 0.05) and also 3 ays after (r = −0.759; *p* < 0.05). The high baseline level of ERFE was accompanied by a high level of HGB (r = −0.718; *p* < 0.05). No significant correlations were found between ERFE and hepcidin.

The analysis showed no significant differences in training load in the study groups (TSS; first week: 1064 ± 54, second week: 1174 ± 37, third week 1238 ± 56—LH-TL group vs. First week: 1131 ± 43, second week: 1181 ± 43, third week 1251 ± 61—NORM group).

## Discussion

Our results revealed a significant decrease in hepcidin level in athletes after 3 weeks of the LH-TL protocol (FiO_2_ = 16.5%, ∼2000 m). The reduced hepcidin was still maintained 72 h after the cessation of the hypoxic stimulus. Hepcidin has a central role in maintaining iron homeostasis. The production of hepcidin is regulated by intracellular and extracellular iron concentrations, erythropoietic iron requirements and inflammation (Ganz and Nemeth, 2012). Hepcidin is regulated by the bone morphogenetic protein/small-body-size mothers against decapentaplegic homolog 1 system (BMP/SMAD), which senses and regulates iron, and the interleukin-6/signal transducers and activators of transcription 3 system (IL-6/STAT3), which is initiated by inflammatory signals (Ogawa et al., 2023). In simple terms, high iron levels and inflammation increase hepcidin transcription. In turn, erythropoietic activity suppresses hepcidin synthesis. In erythroblasts, under the influence of EPO, ERFE secretion is activated, which impairs the production of hepcidin, facilitating the uptake of iron from diet and iron release from macrophage stores (Duarte et al., 2021). The LH-TL protocol in the appropriate dose is considered effective in enhancing erythropoiesis (Płoszczyca et al., 2018); therefore, a lowering of hepcidin was the expected response to our intervention. Our findings are consistent with previous results presented by Govus et al. (2017), who observed that resting plasma hepcidin levels were suppressed after 14 days of LH-TL (FiO_2_ = 15.5%, ∼3000 m) in well-trained endurance athletes. Lowered hepcidin levels allowed for increased availability of iron used to produce hemoglobin during the hypoxic exposure, which is crucial for the effectiveness of altitude training. An increase in the total mass of hemoglobin leads to an increase in blood oxygen-carrying capacity and, consequently, to an improvement in the exercise performance of athletes (Płoszczyca et al., 2018).

Since hepcidin suppression during erythropoiesis is mediated by ERFE (Kautz et al., 2014), we expected that decreased hepcidin levels would be accompanied by increased ERFE. On the contrary, we observed that immediately after LH-TL, ERFE levels were reduced and reached baseline levels after 3 days. Similar results were obtained earlier by Garvican-Levis et al. (2018), who showed that plasma ERFE did not change significantly immediately or 7 days after LH-TL in nonanemic, endurance-trained athletes, despite a downward trend in hepcidin. ERFE expression is regulated by EPO. When the release of EPO stimulates erythrocyte production, it also increases the synthesis of ERFE in bone marrow erythroblasts. In turn, EPO production remains under the control of the HIF-2 factor (Haase, 2013; Srole and Ganz, 2021). It has been shown that after the cessation of hypoxic stimulus, EPO and HIFs return to the pre-exposure levels or temporarily reach levels lower than the baseline (Robach et al., 2004; Lundby et al., 2009; Wiśniewska et al., 2020). The results of our present study indicate that after LH-TL, EPO levels were near baseline and HIF-2α decreased, accompanied by a transient decrease in ERFE levels. This supports the conclusion that the time course of EFRE resembles the kinetics of EPO and HIF changes in response to hypoxia.

The connection between oxygen homeostasis regulated by HIFs and iron metabolism is multi-faceted. HIFs play a key role in cellular adaptation to low oxygen levels. Several HIF target genes are involved in iron homeostasis. Furthermore, HIF activation is modulated by intracellular iron, through regulation of hydroxylase activity. Prolyl hydroxylase domain (PHD) mediates oxygen-dependent degradation of HIF *α* subunit. Under hypoxic conditions, PHD is inactivated and HIF is stabilized. HIF stabilization also occurs at low iron concentrations because PHD is an iron-dependent enzyme and its activity is attenuated when iron concentration is low. It has also been shown that HIF-2α translation is controlled by the activity of iron regulatory protein (IRP), which along with iron-responsive element (IRE), regulates iron absorption across the intestinal epithelium (Anderson et al., 2013; Hirota, 2019; Ogawa et al., 2023). Additionally, some metabolites of the gut microbiota are thought to be able to inhibit the absorption of iron from the intestinal lumen by inhibiting the activation of HIF-2α (Xiao et al., 2023).

HIF is also closely involved in the regulation of proteins and enzymes affecting iron metabolism. First, HIF via ERFE suppresses the production of hepcidin, and promotes Fpn synthesis, the cellular iron exporter located on the vascular side of intestinal cells, which is responsible for delivering iron to the plasma (Ganz and Nemeth, 2012; Ogawa et al., 2023). HIF-2α enhances Fpn synthesis also by direct action (Taylor et al., 2011). Furthermore, HIFs are involved in iron absorption from the intestinal tract by promoting the synthesis of divalent metal transporter 1 (DMT1), duodenal cytochrome b (DCYTB) and hemoxygenase. Studies have shown that HIF also promotes iron transport in the blood and iron uptake into hematopoietic cells through the induction of ceruloplasmin, transferrin (Tf) and transferrin receptor1 (TfR1) (Ogawa et al., 2023). In our study we analyzed changes in HIF-2α and Fpn after completion of a LH-TL protocol. Previous studies indicated that Fpn and HIF-2α increases during high altitude (∼4500 m) exposure (Robach et al., 2007; Goetze et al., 2013). Our research revealed for the first time that after LH-TL protocol, the reduction in HIF-2α levels is accompanied by a decrease in Fpn concentrations. The similar course of changes in HIF-2α and Fpn after LH-TL (reduction) with a simultaneous decrease in the hepcidin level (which should potentially result in a higher concentration of Fpn) may indicate a significant role of the direct effect of HIF-2α on Fpn synthesis during and immediately after hypoxic exposure.

Based on the obtained results, we can conclude that in the first days after the completion of LH-TL, favorable conditions for augmentation of iron availability no longer exist in the body. The cascade of changes in iron metabolism that starts when athletes are exposed to hypoxia stops immediately after hypoxia resolves. Our data show that immediately and 3 days after LH-TL, blood iron levels were not significantly different from baseline. This result is in line with previous studies involving athletes, where serum iron was not altered and remained within the normal range during and after altitude training (Roberts and Smith, 1992; Heinicke et al., 2005; Heikura et al., 2018). From a practical point of view, our results confirm that it is important to ensure sufficient iron stores in athletes’ bodies and to provide the right amount of iron with diet and supplementation before and during altitude training (see: Constantini et al., 2017) in order to make the most of a period of increased erythropoietic activity and facilitated conditions for iron turnover. It should be taken into account that during altitude training, erythroid iron demand increases three-to fivefold (Reynafarje et al., 1959); therefore, supplementation at a dose of even >200 mg elemental iron/day is recommended (Garvican-Lewis et al., 2016; Constantini et al., 2017).

Additional hypoxic stimulation after the end of the LH-TL protocol should also be considered to maintain hematological effects. Our results revealed that immediately after the end of the hypoxic exposure, RBC, HGB and Hct levels were ∼5% higher than baseline, but already 3 days later, they decreased to initial values. Similar changes have been observed in earlier studies on the LH-TL protocol (Robach et al., 2006; Wehrlin et al., 2006; Schuler et al., 2007; Pottgiesser et al., 2012; Czuba et al., 2018). It is assumed that the average life span of erythrocyte is 120 days (Thiagarajan et al., 2021). However, the absence of hypoxic stress after the cessation of hypoxia accelerates neocytolysis, the destruction of the newly formed, youngest circulating RBC within a few days (Alfrey et al., 1997; Risso et al., 2014; Mairbäurl, 2018). It has been suggested that a drop in EPO might be responsible for erythrocyte destruction after removal of the hypoxic stimulus (Rice et al., 2001), so perhaps maintaining elevated serum EPO levels after altitude/hypoxic training might delay the decline in RBC. Recently, hypoxic re-exposure strategies have been proposed in the scientific literature, so as to sustain the hematological adaptations achieved. Pottgiesser et al. (2012) suggested that short-term hypoxia is a potential method to prevent a sudden decrease in EPO after altitude training. Turner et al. (2017) found that 2 h of normobaric hypoxic exposure at ∼4200 m and above is sufficient to increase EPO level. Yan et al. (2021) showed that 3 weeks (12h/day) of hypoxic re-exposure after return from natural altitude training prevented a drop in EPO and maintained the hematological adaptations in athletes over this period, whereas in subjects without additional hypoxic stimulus, Hb_mass_ gain was lost 9 days after the intervention. The effectiveness of the hypoxia re-exposure strategy after the LH-TL protocol is unknown and this issue is worth considering in future research.

The fact that in our study the level of hepcidin maintained a downward trend until the third day after the end of hypoxia, despite the decrease in HIF-2, EPO and ERFE, may seem puzzling. It should be noted, however, that several previous studies have suggested a negative relationship between HGB levels and hepcidin (Uehata et al., 2012; Mercadal et al., 2014; Pan et al., 2016). Our results confirmed this relationship. In the group exposed to hypoxia, a high level of HGB immediately after LH-TL was accompanied by a low level of hepcidin both directly (r = −0.749; *p* < 0.05) and on the third day after the cessation of hypoxia (r = −0.759; *p* < 0.05). Secondly, ERFE is most probably not the only factor that suppresses hepcidin expression in response to hypoxic conditions. It is thought that hepcidin inhibition might occur through an interplay between factors directly activated by hypoxia and factors activated through hypoxia-induced erythropoiesis (Ravasi et al., 2014). It is proposed that, in addition to ERFE, other important regulators of hepcidin response to hypoxia are platelet-derived growth factor (PDGF), growth differentiation factor 15 (GDF15) and twisted gastrulation (TWSG1) (Tanno et al., 2009; Gassmann and Muckenthaler, 2015). Previous research has shown that in a hypoxic environment there is an increase in GDF15 and PDGF, accompanied by a decrease in hepcidin levels (Piperno et al., 2011; Talbot et al., 2012; Goetze et al., 2013; Altamura et al., 2015). Using correlation analysis, Sonnweber et al. (2014) found that PDGF is associated with hypoxia mediated hepcidin repression in humans. Interestingly, Zembroń-Łacny et al. (2020) recently showed that PDGF remained significantly higher 1 day after the passive 12-day intermittent hypoxic exposure, suggesting that PDGF could potentially have contributed to maintaining the downregulation of hepcidin observed in our study. However, we did not measure PDFG’s response to the LH-TL protocol–this is worth considering for future research. On the other hand, it is also suggested that PDGF and ERFE might be major factors involved only in the early phase of hepcidin suppression by hypoxia, whereas alternative but not yet well recognized mechanisms are proposed to regulate the later stages of hepcidin downregulation under hypoxic conditions (Ravasi et al., 2014; Sim et al., 2019). It should also be taken into account that, in our study, despite statistical significance, the differences between hepcidin levels before and after LH-TL were small or even trivial (ES: ≤0.21); therefore, the observed drop in hepcidin may not be of practical relevance. Thus this result should be considered with caution.

Our research also provides data on the response of hepcidin and ERFE to 3 weeks of normoxic training. Previous studies indicate that hepcidin increases in response to acute endurance exercise (Peeling et al., 2014; Larsuphrom and Latunde-Dada, 2021). However, currently there is little data on the cumulative effects of regularly performed exercise training on the expression of iron regulatory factors. Our results revealed that hepcidin increased after 3 weeks of training in normoxia. This is in line with results from Ishibashi et al. (2017), who observed elevated hepcidin during an intensified training period in female long-distance runners. Contrarily, Moretti et al. (2018) demonstrated the reduction of hepcidin levels after 3 weeks of exercise training in recreational male runners. The increase in hepcidin levels under the influence of exercise in normoxia is mainly explained in terms of inflammation with increases in IL-6 (Ruchala and Nemeth, 2014) and it depends on the training intensity and adaptation to training loads. Zügel et al. (2020) found that resting hepcidin concentrations were elevated in rowers at the beginning of the high load training phase and returned to baseline within a few days of training camp. The authors conclude that the return of hepcidin to the baseline reflected an adaptation to high workloads in rowers. It is possible that in our study, the increasing training workload in the following weeks of intervention did not allow adaptation to be achieved, hence the persistently high level of hepcidin.

It has previously been found that after 3 weeks of exercise training in normoxia, ERFE remained at an unchanged low level in male runners and it did not correlate with hepcidin levels (Moretti et al., 2018). Our results confirmed these reports: in our study, ERFE was not affected by 3 weeks of training in normoxia and there was no significant relationship between ERFE and hepcidin. Additional research is likely to provide more clarification on the biological role of ERFE for iron status and its association with hepcidin under training conditions.

Finally, it is worth noting the reverse trend of changes in hepcidin in normoxic and hypoxic conditions, which we demonstrated in our study. Hepcidin levels increased in the group that trained and stayed in normoxia, possibly due to an increase in inflammation induced by exercise workloads (Peeling et al., 2014; Domínguez et al., 2018). Factors involved in inflammation, such as IL-6, act as a signaling molecules for hepcidin synthesis, causing functional iron deficiency, inhibition of EPO production and suppresses erythropoiesis (Vyoral and Petrák, 2005; Eisenstaedt et al., 2006; Icardi et al., 2013). In contrast to inflammation, hypoxia is a potent suppressor of hepcidin and promotes increased transport of iron from its storage sites, which facilitates erythropoiesis in the bone marrow (del Campo and Zamarrón, 2015; Kiers et al., 2019). Kiers et al. (2019) demonstrated that hypoxia partially attenuates the inflammation-induced increase in hepcidin levels and mitigates the decline in serum iron, likely through increased synthesis of EPO and ERFE. Our data partially confirm these reports. In the group of athletes exposed to hypoxia, hepcidin levels decreased, despite the fact that the training program was identical as for the normoxic group, and so exercise-induced inflammation probably was similar. This finding suggests that the effect of hypoxia on hepcidin regulation during altitude training had a dominant role over the influence of pro-inflammatory cytokines, which were increased by exercise-induced inflammation. However, in this study we did not analyze the indicators of the inflammatory response, so the above relationship is conjectural. Future studies should consider assessing the relationship between hypoxia, inflammation, hepcidin and ERFE in athletes, because it may be of practical importance not only for the effectiveness of altitude training, but also for athletes whose regular physical exercise leads to a decrease in iron stores.

## Limitations

Our study has several limitations. First, due to the nature of the intervention, we could not conduct the study with a crossover design, so we cannot completely rule out the possibility of a confounding effect of individual response. Secondly, we did not determine ferritin concentration in the subjects before the experiment. It is possible that, much like what happens in response to acute exercise, an athlete’s iron stores may influence the baseline hepcidin levels and the magnitude of post-exercise hepcidin response. Low serum ferritin levels are associated with the absence of hepcidin response to exercise (Peeling et al., 2014; Tomczyk et al., 2020). In our study, for all pre- and post-intervention athletes, serum iron was in the normal range, but in future research, serum ferritin should be included in the measurements. Similarly, it would also be helpful to determine the level of transferrin as an indicator of iron availability and the concentration of soluble transferrin receptor (sTfR), which enables the detection of a recent high erythropoietic activity at a time when EPO is blunted (Robach et al., 2004), as in our study after completion of the LH-TL protocol. For a complete view of changes in iron regulatory factors, additional measurements would be advisable during the LH-TL procedure (for example, after 1, 3, 7 and 14 days of exposure), and not only after its completion. However, for organizational reasons, we did not have such an opportunity. We relied on knowledge of changes in hepcidin and ERFE concentrations in the early stage of hypoxic exposure from studies conducted by other authors (Talbot et al., 2012; Goetze et al., 2013; Govus et al., 2017; Garvican-Lewis et al., 2018). Finally, our subjects had an average level of fitness (VO_2max_ ∼56 mL kg^-1^∙min^-1^), thus our results should not be extrapolated to elite athletes or untrained subjects, nor to iron-deficient athletes or female athletes.

## Conclusion

In conclusion, we found that 3 weeks of normobaric hypoxic exposure to 2000 m simulated altitude by 11–12h/day (LH-TL protocol) suppresses resting hepcidin and ERFE levels in trained endurance athletes. Three days after cessation of hypoxia, hepcidin remains at a reduced level, but ERFE returns to baseline. Our results indicate that ERFE is probably not the only factor that suppresses hepcidin expression in response to moderate hypoxic conditions, especially in later stages of hepcidin downregulation. After the cessation of the hypoxic stimulus, the favorable conditions for augmentation of iron availability used to produce functional hemoglobin during erythropoiesis are no longer present (decreased ERFE, Fpn and HIF-2α). Three days after LH-TL protocol, hematologic variables that increased immediately after the intervention (RBC, HGB and Hct) decreased to baseline values. From a practical point of view, it seems worth considering the introduction of additional hypoxic stimulation after the LH-TL protocol to maintain hematological effects in athletes. In addition, our results revealed an inverse trend of changes in hepcidin levels after the implementation of the training program with and without exposure to hypoxia. This finding points to the probable dominant role of erythropoietic activity caused by hypoxia exposure over the effect of inflammation on hepcidin concentration changes.

## Data Availability

The raw data supporting the conclusion of this article will be made available by the authors, without undue reservation.
